# Basement membranes’ role in immune cell recruitment to the central nervous system

**DOI:** 10.1186/s12950-024-00426-6

**Published:** 2024-12-20

**Authors:** Shaun A. Wright, Rachel Lennon, Andrew D. Greenhalgh

**Affiliations:** 1 Lydia Becker Institute of Immunology and Inflammation, Division, Division of Immunology, Immunity to Infection and Respiratory Medicine, School of Biological Science, Faculty of Biology, Medicine and Health, The University of Manchester, Manchester, UK; 2 Cell Matrix Biology & Regenerative Medicine and Wellcome Centre for Cell-Matrix Research, School of Biological Science, Faculty of Biology, Medicine and Health, The University of Manchester, Manchester, UK; 3 The University of Manchester, Oxford Road, Manchester, M13 9PT UK

**Keywords:** Central nervous system, Brain, Basement membrane, Extracellular matrix, Leukocyte extravasation, Neuroinflammation, Blood-brain barrier

## Abstract

Basement membranes form part of the extracellular matrix (ECM), which is the structural basis for all tissue. Basement membranes are cell-adherent sheets found between cells and vascular endothelia, including those of the central nervous system (CNS). There is exceptional regional specialisation of these structures, both in tissue organisation and regulation of tissue-specific cellular processes. Due to their location, basement membranes perform a key role in immune cell trafficking and therefore are important in inflammatory processes causing or resulting from CNS disease and injury. This review will describe basement membranes in detail, with special focus on the brain. We will cover how genetic changes drive brain pathology, describe basement membranes’ role in immune cell recruitment and how they respond to various brain diseases. Understanding how basement membranes form the junction between the immune and central nervous systems will be a major advance in understanding brain disease.

## Basement membranes – specialised extracellular matrix

**Fig. 1:**
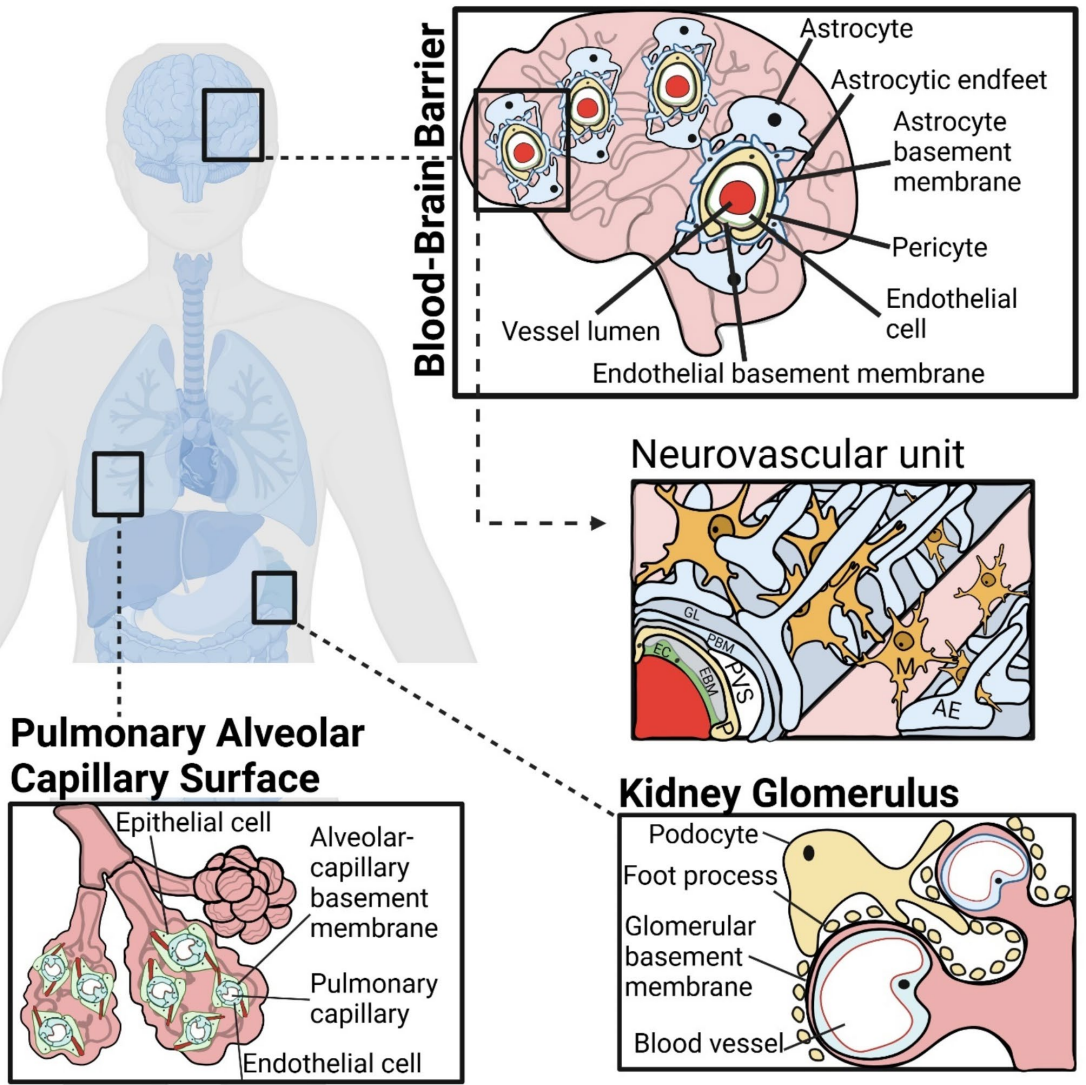
Schematic shows specialised basement membranes in different regions of the human body. Examples include the parenchymal and endothelial basement membrane of the blood-brain barrier, the alveolar-capillary basement membrane of the pulmonary alveolar capillary surface, and the glomerular basement membrane of the kidney glomerulus. Also shown is the location of the parenchymal-, and endothelial basement membrane, with reference to the neurovascular unit within a penetrating arteriole. EC = Endothelial cell, EBM = Endothelial basement membrane, P = Pericyte, PVS = Perivascular space, PBM = Parenchymal basement membrane, GL = Glial limitans, AE = Astrocytic end feet, M = Microglia

The extracellular matrix (ECM) makes up over a third of body mass and forms the scaffolds and structural basis of tissues [1]. Extracellular matrix is arranged as two discrete entities: either basement membranes or interstitial matrices, and it exists in close association with the endothelial glycocalyx [2, 3]. It consists of polymeric protein networks and fibrils, which perform essential functional roles in tissue organisation and remodelling alongside the regulation of cellular processes [4, 5]. The mechanisms used to lay down extracellular matrix proteins are highly conserved across the eukaryotes and likely existed prior to the emergence of the metazoan [6]. The biology of basement membranes and interstitial matrix is intertwined, and they both may control cell proliferation, differentiation, angiogenesis and homeostasis [7–10]. Basement membranes are discrete from interstitial matrices in that they are thin, cell-adherent sheets of ECM which underlie all continuous sheets of cells, ranging from vascular endothelia to Schwann cells, thereby surrounding and supporting most tissues [11]. Additionally, they constitute functionally important structures at key interfaces, such as in the kidney glomerulus, the blood-brain barrier (BBB) and pulmonary alveolar capillary surface and play critical roles defining boundaries of tissues and protecting developing tissues from mechanical damage (Fig. 1) [12]. Interstitial matrix comprises the ECM components found between cellular structures [13] and have more plastic roles, forming the basis of various tissue environments, from gel-like scaffolds to dense tendons. In doing so, interstitial matrices facilitate the adoption of cellular environments conducive to basic biology, from inter-cell interactions to signalling [14]. This review will focus on basement membrane biology in the context of the central nervous system (CNS).

While basement membranes are distributed throughout the body they have diverse protein composition across organ- and tissue-sites, likely reflecting their varied and specialised roles [15]. Basement membranes have essential core components including type IV collagen, laminins, nidogen and heparan sulfate proteoglycans (HSPGs) [15]. Laminins are the most abundant non-collagenous basement membrane protein and networks of laminin are considered the foundation template of all basement membranes [16–18]. Three subclasses of laminin chain isoforms exist: five *α*-, four *β***-**, and three *γ*- chains, and their appropriate assembly forms one of 16 different laminin heterotrimers, Table 1 [19–21]. Laminins of the vascular basement membrane consist of either α1, α2, α4or α5 combined with *β* 1 and *γ* 1 chains, giving rise to laminin isoforms: 111, 211, 411 and 511 (see Table 1) [22]. Laminin 421 is also found in the cerebral vasculature [16, 23, 24]. In the CNS, the interaction of laminin *α−*chains and integrins expressed by brain capillary endothelial cells, pericytes and astrocytes supports the anchoring of laminin to integrin- and dystroglycan-receptors, thereby producing a laminin-cytoskeleton macromolecular structure [20, 23, 25].

**Table 1:** Laminin isoform naming system

Alpha chain	Beta chain	Gamma chain	Heterotrimeric form of laminin
α1	β1	γ1	Laminin 111
α2	β1	γ1	Laminin 211
α4	β1	γ1	Laminin 411

There are 28 collagens in the mammalian genome and type IV collagen is the most abundant in basement membranes. At a fundamental level, collagen confers tissue strength and resilience [1]. Heterotrimeric polypeptide chains of type IV collagen exist in three forms: collagen IV α1α1α2, collagen IV α3α4α5and collagen IV α4α5α6 [26, 27]. The most abundant collagen IV isoform in the brain parenchyma is collagen IV α1α1α2 [28–31]. Networks of laminin and collagen IV are joined together by nidogen, which exists in two forms: nidogen-1 and nidogen-2 [29]. Nidogen-1 plays a fundamental role in supporting and joining the self-assembled layers of laminin and collagen IV while also binding fibulin and perlecan [32–35]. Whereas nidogen-2 transcripts demonstrate significant downregulation at early postnatal ages, evidence of the role it appears to play in embryogenesis [36].

The vascular basement membrane also contains three types of heparan sulfate proteoglycans: perlecan, agrin and collagen XVII. Perlecan is the most abundant heparan sulfate proteoglycan, and it contains a multidomain protein core that has three glycosaminoglycan (GAG) chains at its N-terminus. It is involved in maintaining the integrity of the basement membrane and binding of growth factors and is found within the mature vascular basement membrane [37, 38]. Moreover, the HSPGs found in the vascular basement membrane harbour growth factors that protect against degradation and may affect paracrine signalling of the surrounding cells [39, 40]. Alongside these proteins are matricellular proteins, those which have additional functions beyond basement membrane structure and assembly [41]. Studies characterising the broader composition of the ECM, support the existence of over 1000 matrisome proteins [42]. In addition, secreted regulatory proteins, such as proteinases and chemokines are often found in association with core basement membrane components [15]. The sum of the functions of the different basement membrane-components give rise to a complex signalling platform with which cells interact.

### Core basement membrane components: lessons from genetics

Genetic tools to investigate the function of core basement membrane proteins are revealing insights into their biology and function (Table 2). Heterozygous, semi-dominant pathogenic *COL4A1* and *COL4A2* variants exist in humans, and they are appropriately modelled by mouse lines [31, 43–45]. Pathologies have been recorded in all organs so-far examined when assessing the phenotype of *Col4a1* and *Col4a2* mutant mice, reflecting the almost ubiquitous distribution of COL4A1 and COL4A2 in all basement membranes [45]. Variants of *COL4A1* and *COL4A2* in humans produce systemic disorders with implications most often reported in the vasculature, brain, eyes, kidneys and muscles [45–47]. Cerebrovascular disease, which regularly manifests as porencephaly, small-vessel disease and recurrent intracerebral haemorrhages, is associated with mutated *COL4A1* and *COL4A2* mutations [48]. In mice, the global knockout of the collagen IV genes *Col4a1* and *Col4a2* (Col4a1^−/−^; Col4a2^−/−^) gives rise to an atypical basement membrane and embryonic lethality at E10.5-E11.5, but not before E9.5. The double homozygotes exhibit atypical capillary network throughout angiogenesis, impaired placental development and neuronal ectopias [49]. Hence, *Col4a1* and *Col4a2* are required for basement membrane homeostasis but not for its initial construction [49, 50]. Conversely, double heterozygote mice for *Col4a1* and *Col4a2* null alleles (Col4a1^+/−^;Col4a2^+/^) are viable and lack any overt phenotype. However, heterozygous mice for missense or splicing mutations in *Col4a1* and *Col4a2* produce complex pleiotropic phenotypes, including ocular, central nervous, nephropathic and reproductive malformations [31, 51]. Tissue specific manipulation can provide more focussed analysis of COL4A1 and COL4A2 within brain cell types and the ablation of exon 41 of *Col4a1* in astrocytes caused mild intracerebral haemorrhage. The same mutation in brain microvascular endothelial cells or pericytes produced a fully penetrant intracerebral haemorrhage and incompletely penetrant porencephaly phenotype [52]. While mice with biallelic splice mutations of exon 41 of *Col4a1* are not viable, offspring with one mutated allele exhibit porencephaly and intracerebral haemorrhage which presents as fully penetrant multi-focal and recurrent intracerebral haemorrhage as adults [31, 43, 52]. Importantly, reductions in collagen IV α1α1α2 subunit expression have previously been associated with porencephaly and haemorrhagic stroke in humans [31, 43, 53].

Despite this, mutagenesis screens represent invaluable resources to assess the contribution of individual chains of collagen IV in relation to CNS homeostasis and we predict that more variants in *COL4A1* and *COL4A2* will be linked to abnormalities of CNS biology. To this end, the generation of mice which mirror human variants in *Col4a1* and *Col4a2* associated with abnormalities of the CNS is required.

**Table 2:** Shown are various mutations in mice in which core basement membrane proteins are knocked out, along with the associated phenotypes

Protein	CNS-resident cell expression	Mutation	Cre lines	Phenotypes	Ref.
Collagen 4A1	Endothelial cells Astrocytes Mesenchymal cells Pericytes [54–57]	Biallelic Δexon41 Monoallelic Δexon41 Conditional knockout	Tie2-Cre PDGFRβ-Cre GFAP-Cre	Embryonic lethality, intracerebral haemorrhage Perinatal lethality with intracerebral haemorrhage, porencephaly Intracerebral haemorrhage porencephaly Intracerebral haemorrhage porencephaly Mild intracerebral haemorrhage	[53] [31, 43] [52]
Collagen 4A1/4A2	Endothelial cells Astrocytes Mesenchymal cells Pericytes [54–57]	Missense mutations		Varying degrees of brain damage	[51, 53, 58]
Laminin α2	Astrocytes Oligodendrocytes Endothelial cells [59, 60]	Global null mutation		BBB disruption	[61, 62]
Laminin α4	Endothelial cells Oligodendrocyte precursor cells [60, 63]	Global null mutation		Perinatal haemorrhage	[64]
Laminin γ1	Astrocytes Endothelial cells Oligodendrocyte precursor cells [63, 65, 66]	Conditional knockout	Nestin-Cre PDGFRβ-Cre	BBB breakdown, intracerebral haemorrhage BBB breakdown and hydrocephalus on C57Bl6/FVB hybrid background Age-related mild BBB breakdown in C57BL6 background	[61, 67]

The contribution of laminin α5 [68–70], *β* 1 [17] and *γ* 1 [17, 71, 72] to BBB integrity have proven difficult to investigate given that their global knockout produces non-viable embryos [17, 71, 73–76]. As a result, cell-specific conditional knockout mouse lines targeting individual laminin chains have been generated [50]. The loss of laminin-211, astrocyte-derived laminin, give rise to viable offspring which exhibit age-dependent BBB breakdown and intracerebral haemorrhage [61]. Whereas, laminin α2 mutants exhibited postnatal BBB disruption, suggesting that astrocytic laminin performs a critical role in BBB maintenance [62]. Mural cell specific laminin deficient mice have BBB breakdown and hydrocephalus and rarely survived past four months of age [77]. Critically, these deficits are not found when laminin-511, vascular smooth-muscle cell-derived laminin, is genetically ablated, highlighting the importance of laminin-511 in maintaining BBB integrity in smooth muscle cells [77, 78]. However, given that hydrocephalus itself can compromise BBB integrity, it remains unclear whether BBB breakdown is a direct result of the loss of basement membrane laminin, or is a secondary insult following hydrocephalus [78]. Laminin α4 null mutants are viable, but they do exhibit compromised vascular integrity and haemorrhage at perinatal stage [64]. Laminin α5 expression in the vasculature begins following birth and it is thought that laminin α5 expression may rescue the loss of laminin α4 within the vasculature [79, 80]. Indeed, mice lacking laminin α5 do not present an overt phenotype in homeostasis [81, 82]. Clearly, laminins are important players in vascular basement membrane biology in the context of CNS, but compensatory mechanisms between discrete chain isoforms have made their precise roles difficult to disentangle. Human variants of laminin are associated with diseases, including laminin a2-related muscular dystrophy. While the loss of laminin-a2 primarily causes skeletal muscle damage, phenotypes have been recorded in the peripheral nerve and the brain [83]. In the brain, *LAMA2* muscular dystrophy patients display changes in white matter density, as may be seen through T2-weighted magnetic resonance, cerebellar hypoplasia, and less frequently, occipital lobe neuronal migration defects [84–87]. A subset of patients have also been shown to demonstrate moderate cognitive complications and they also exhibit seizures [88]. Other human diseases linked to genetic variants in laminin include: junctional epidermolysis bullosa and Pierson syndrome, involving laminin-332 and laminin-521, respectively [89, 90]. Genetic variants in laminin-332 and laminin-521 are not typically associated with abnormalities of the CNS. Despite this, a study employing a *LAMB2* knockout mouse demonstrated a disrupted pial basement membrane and an ectopic distribution of cortical plate cells alongside atypical radial glial cell development [91, 92]. Extrarenal manifestations of *LAMB2* mutations have been reported in patients that survive infancy including, neurodevelopmental deficits with delayed motor and cognitive development, hypotonia and hearing deficits [93, 94]. Additionally, a report details a patient with gross and microscopic CNS abnormalities, including cortical hypercellularity and disorganisation, alongside disorganisation of neurons involving the hippocampus and dentate nucleus of the cerebellum [95].

The role of nidogen and perlecan is also critical to consider in the context of the biology of the CNS, given their ubiquitous expression in all basement membranes [96]. The knockout of nidogen-1 in mice produces mild alterations in brain capillary basement membranes [33, 76, 97] while mice with nidogen-2 knockouts do not have a phenotype [98]. And, while nidogen-1 expression is not changed in nidogen-2 null mice [98], nidogen-1 knockout mice exhibit significant redistribution and upregulation of nidogen-2 [99]. The knockout of both nidogen-1 and nidogen-2 leads to severe basement membrane defects and perinatal lethality [100–102], highlighting a likely compensatory mechanism between nidogen-1 and nidogen-2. To this end, generating nidogen-1 and nidogen-2 conditional knockout models specifically in the CNS-supporting cells would provide critical insights into the role that nidogens perform in the CNS. The global knockout of perlecan produces non-viable offspring that die between E10-E12 [103]. The perlecan null mice exhibit impairments in brain development but typical basement membrane structure [104]. Studies utilising conditional perlecan-deficient mice show that perlecan is not required for the assembly of the basement membrane or for homeostasis of the BBB [105]. Despite this, there are complex phenotypes involving cartilage and the lens capsule, suggesting that perlecan is required for embryogenesis but not basement membrane formation [103, 106, 107]. The early embryonic lethality associated with the global knockout of perlecan makes it difficult to assess the role that perlecan performs in the CNS.

The contribution of individual components of the basement membrane to CNS biology likely remains underappreciated, due largely to the intrinsic complexity of the basement membrane [78]. As such, the exact mechanisms through which the basement membrane, and its constituent components, regulate homeostasis of the CNS at the molecular- and cellular-level remain unexplored. Given the involvement of many basement membrane components in pathologies affecting the CNS, increasing understanding of the relationship between the basement membrane and the disorders affecting the CNS would likely prove useful in the development of therapies.

## Basement membranes in the CNS during homeostasis

As loss, mutation or manipulation of basement membrane genes has shown, these structures are equally fundamental in the brain as anywhere in the body [78]. The cerebral vasculature tree contains distinct forms of blood vessels, including the major arteries that penetrate the pial surface, arterioles, capillaries, postcapillary venules, venules and veins [108]. Two discrete basement membranes are found in association with the vessels in the brain, the endothelial basement membrane and the parenchymal basement membrane [109]. Pericytes and endothelial cells of the secrete laminin-111 and − 112 and, in conjunction with vascular tight-junction proteins, act to restrict paracellular diffusion. The parenchymal basement membrane is composed of astrocyte-derived laminin-411 and − 511 and it forms a discrete unit adjacent to the endothelial basement membrane, in all vessels other than cerebral capillaries, separated by a perivascular space at post-capillary venules. Astrocytes around the parenchymal basement membrane make contact with the underlying basement membrane with their end-feet, and form a continuous layer called the glia limitans [110]. In the homeostatic brain, the astrocytic- and the pial-basement membrane appear as a single structure when visualised using light microscopy within capillaries [111].

The linkage between the parenchymal- and endothelial basement membrane within the capillary regions of the BBB is a site of nutrient transfer, however the mechanisms that govern its fusion are unknown [111, 112]. Investigations of fusion between adjacent basement membranes, such as the the endothelial and podocyte basement membranes that fuse to form the glomerular basement membrane and the alveolar basement membrane, which fuses in regions with the vascular basement membrane suggest that fused basement membranes are important at organ-blood barriers [113, 114]. Research investigating the pathogenesis of experimental autoimmune encephalomyelitis (EAE) demonstrated that the fused basement membranes in the capillaries of the brain prevent the appropriate trafficking of T cells. However, mononuclear cells may infiltrate the brain parenchyma through the perivascular space, the region which separates the two basement membranes within post-capillary venules of the CNS [111]. Generation of the specialised connection between adjacent basement membranes of the blood vessels endothelial cells and the neuronal cells in the brain is poorly understood but these connections may have important functions in regulating immune cell recruitment to the CNS. However, probing the mechanisms required to establish this important interface have proven difficult, given the complex interactions that exist between cell types, basement membrane components and tissue function [115]. Evidence from other basement membrane connections suggests that *fibulin-1* and *hemicentin-1* are necessary, but no knockout studies using FBN1/HMCN1 null mice focussing on the brain have been published [16, 116, 117]. Given the importance of this structure in the biology of other blood-organ barrier sites, it remains likely that its role in blood-brain barrier is currently underappreciated.

**Fig. 2:**
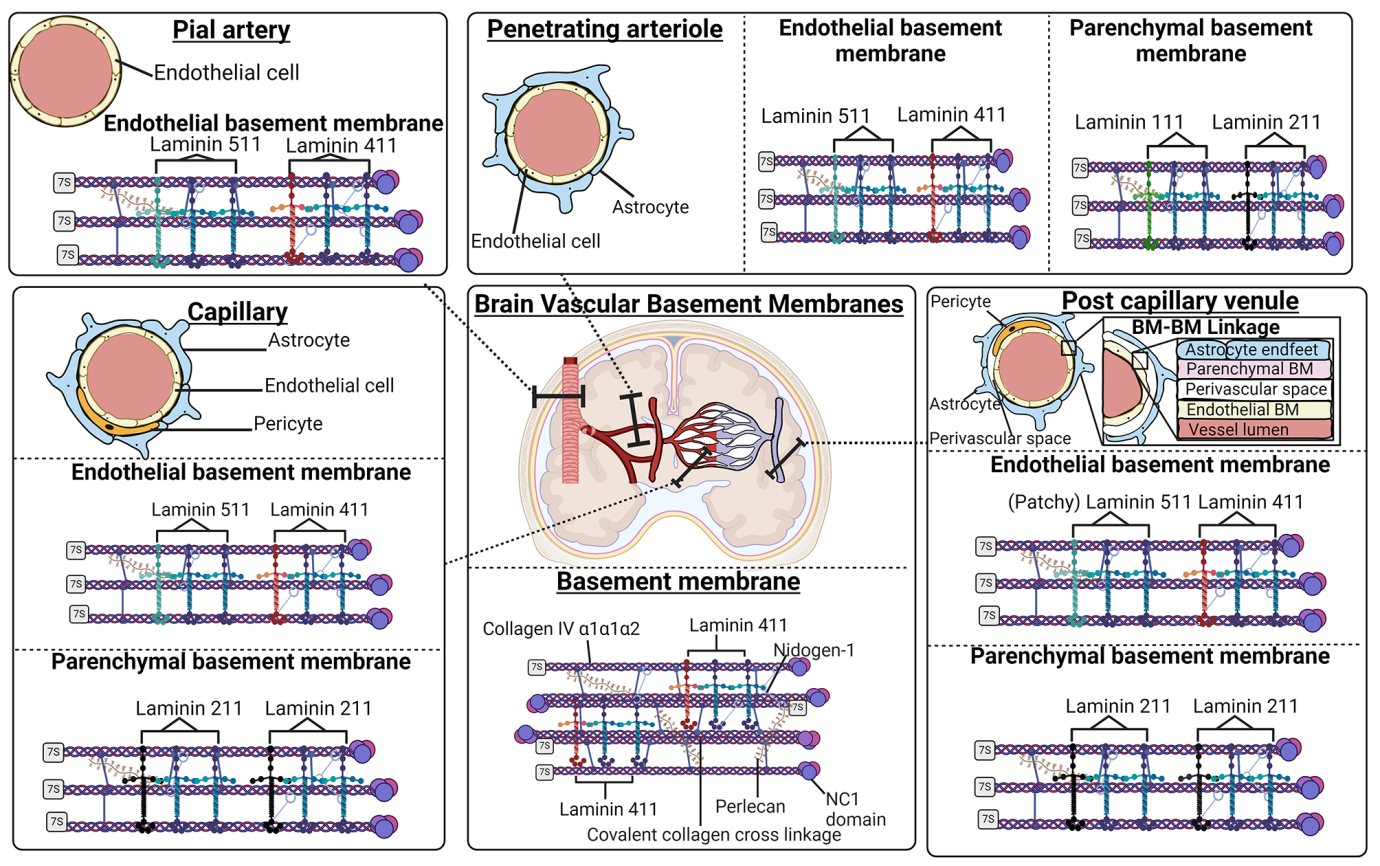
Simplified schematic of the vascular tree in the central nervous system shows the varied expression of laminin chain isoforms between pial arteries, penetrating arterioles, capillaries and postcapillary venules. A simplified schematic of the BM-BM linkage is shown for visualisation purposes but note that it is not biologically accurate; given that the parenchymal and endothelial BM may make contact. A general basement membrane is given to exhibit the core basement membrane components

The composition of basement membranes varies in different vessel walls (summarised in Fig. 2) [22, 118, 119]. It is interesting to note that, within penetrating arterioles, two separate basement membranes exist, separated by a perivascular space [120]. Intriguingly, it is within these spaces that perivascular macrophages (PVMs) and other cells may be found and that leukocytes accumulate in cerebral inflammation prior to their infiltration into the brain [121, 122]. Another important point is that the absence of laminin 111 distinguishes capillaries from arterioles and can be explained by the lack of contributing pial cells in the cerebral capillaries. The endothelial basement membrane of the postcapillary venules contains an irregular distribution of laminin 511, which has previously been associated with the failure of leukocyte extravasation [111]. Disruption to the integrity of the basement membrane is an important event during BBB dysfunction and the subsequent infiltration of leukocytes into the surrounding neural tissue is observed in many neurological conditions [78].

The brain has other anatomically and functionally important interfaces with the periphery, such as the choroid plexus (CP) and the meninges. In recent years these regions have become the focus of considerable research efforts because of the diverse immune cell populations now known to reside within these regions [123]. The choroid plexus is present in all four ventricles of the brain and its constituent monolayer of polarised secretory epithelial cells secrete 60–75% of cerebrospinal fluid (CSF) [124]. The subendothelial basement membrane of the murine choroid plexus is reported to contain collagen IV α1α1α2 whereas the subependymal basement membrane has been reported to contain α3α4α5 [125]. Intriguingly, while collagen IV α1α1α2 heterotrimers are widely distributed across all basement membranes, collagen IV α3α4α5 heterotrimers have previously been reported only in the basement membrane of the glomeruli of the kidney, alveoli of the lungs, testis, inner ear cochlea and eyes [126–132]. Another specialised immune interface of the brain is the meninges, specialised functional layers, including the pia-, the arachnoid- and the dura-mater, which collectively ensheathe the brain parenchyma and the spinal cord [133]. The meninges are immune cell hubs, which perform a broad range of roles [133]. Immunolocalisation of murine pia mater has previously demonstrated the existence of two discrete heterotrimeric forms of collagen IV, collagen IV α1α1α2 as well as collagen IV α5α5α6 [125]. Collagen IV α5α5α6 heterotrimers have previously been documented in the basement membranes of bronchial epithelium, smooth muscle cells of the bladder, uterus, stomach, oesophagus, small intestine, skin, and Bowman’s capsule and tubules of the kidney [126]. Despite the observations of collagen IV α3α4α5 and α5α5α6 there exists a lack of evidence from studies employing human tissue. To this end, thorough characterisations of the localisation of individual subtypes of type IV collagen are required. Clearly, there remains inconsistencies in our understanding of the composition and function of the varying basement membranes in, and around, the CNS. Despite this, the existence of a fused basement membrane within the BBB is reminiscent of the fused basement membrane of the kidney. Whether the mechanisms that govern the fusing of these basement membranes, or their maintenance, are conserved or not are important questions given the functional importance of both structures and their involvement in a broad range of, potentially fatal diseases. Indeed, research may well benefit from investigating the similarities between the two fused basement membranes to inform our understanding. Moreover, thorough characterisations of basement membrane proteins, in and around the brain parenchyma, are necessary to aid in research aimed at ameliorating the symptoms of disease that affect the CNS.

## Basement membranes as a regulator of leukocyte extravasation

Leukocytes can be rapidly recruited to sites of inflammation from the blood [134]. Endothelial cells line vascular walls and form an almost continuous barrier which leukocytes must cross during extravasation [135]. Leukocyte–endothelial interactions and extravasation to tissue beds most commonly occur in postcapillary venules but can also take place in large veins, capillaries, and arterioles [136]. The vascular basement membrane is critical for blood vessel homeostasis, where it acts as a semi-permeable barrier that dictates the passage of leukocytes into surrounding regions [137, 138]. Endothelial cells and pericytes actively synthesise venular basement membrane components [139]. Importantly, the molecular composition of the venular basement membrane is discrete in that the major laminin isoforms found within it are laminin-411 and laminin-511 [140]. However, it has proven difficult to study the process of cell migration across basement membranes due to an inability to appropriately reproduce their complexity in vitro [141].

The migration of leukocytes from the vascular system of the CNS has been studied in various pathologies, such as stroke. Blood vessels of the CNS that form the BBB form an extremely tight endothelial layer, through their tight junctions [142]. The recruitment, migration to inflammatory areas, and targeting of leukocytes is caused by stimulation and expression of adhesion molecules, such as intracellular adhesion molecule 1/2 (ICAM-1/2) and vascular cell adhesion molecule 1 (VCAM-1) [143]. ICAM-1 and ICAM-2 are expressed on the surface of various populations of leukocytes, endothelial cells and platelets [144–147], whereas, fibroblasts, epithelial cells and pericytes do not express ICAM-2 [144].

**Fig. 3:**
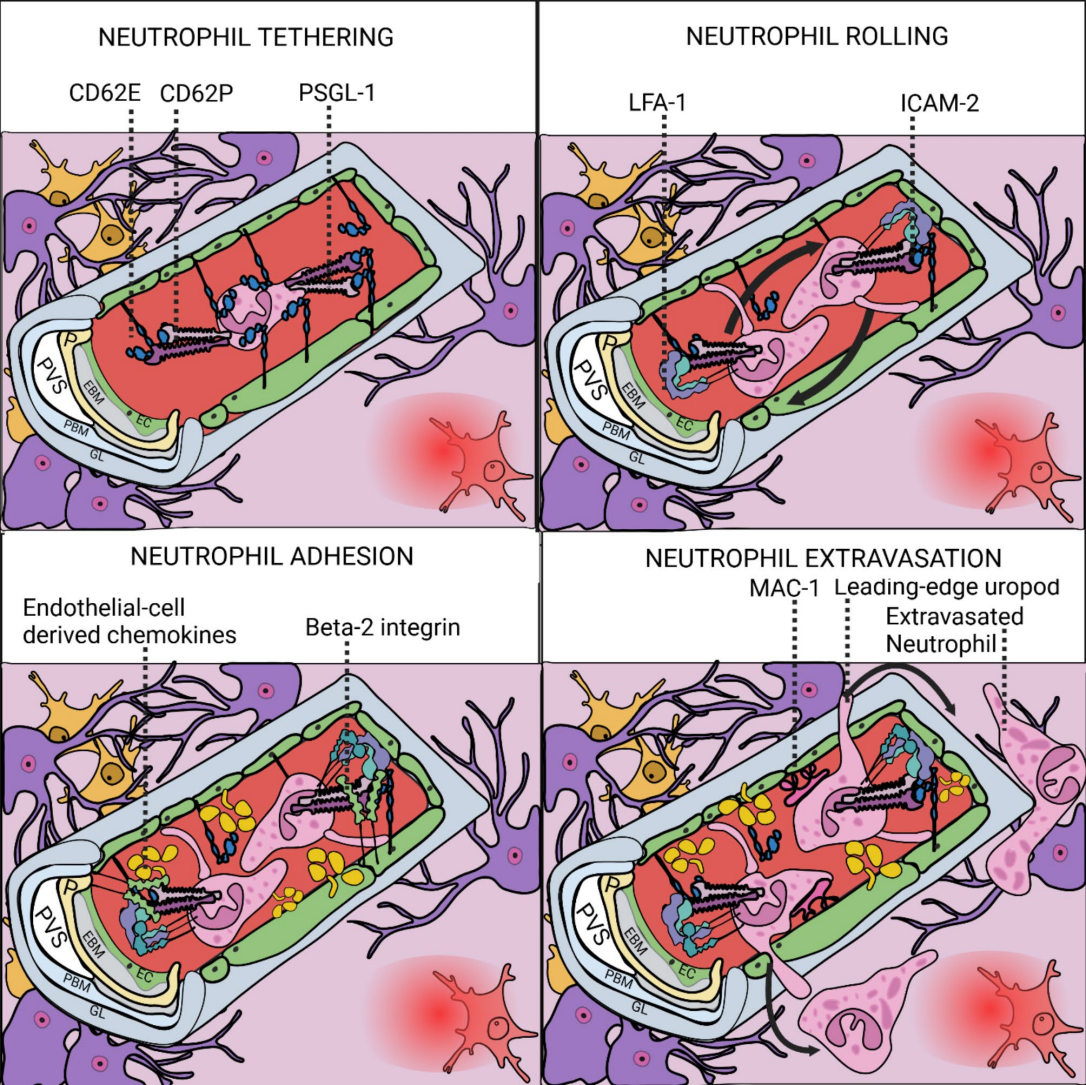
Simplified diagram showing the process of neutrophil vessel extravasation, split into four panels: Tethering, Rolling, Adhesion and Extravasation. During neutrophil tethering, endothelial CD62 interacts with CD62P and PSGL1 establishing a transient contact. In rolling, LFA-1 and ICAM-2 react in trans and allow neutrophils to roll. In adhesion, endothelial-cell derived chemokines induce the expression of beta-2 integrin, which induces neutrophil adhesion/arrest. Finally, neutrophils ‘crawl’ along blood vessels in a MAC-1 dependent manner and use their leading-edge uropod to extravasate out of blood vessels. Illustrations of the following cells are shown: astrocytes in purple, microglia in yellow and activated microglia in red

Transendothelial cell migration (TEM) from the circulation across the inflamed post-capillary venule is a multistep process (summarised in Fig. 3). Initial tethering and rolling of neutrophils is followed by slow rolling, activation of integrins, shear resistant arrest, crawling, diapedesis, and the eventual migration across the pericyte layer [148, 149]. Endothelial (E)-selectin, CD62E, on the endothelial surface interacts with platelet (P)-selectin (CD62P) and P-selectin glycoprotein ligand (PSGL)-1 on the leukocytes to initiate an initial transient contact between a circulating cells and the inflamed vascular post-capillary venule [150–152]. While rolling, leukocytes interact with endothelial cell-presented chemokines which initiate the affinity maturation integrins, which permits the arrest to the endothelium [153–155]. Once arrested, cells crawl of the luminal surface of endothelial cells until they reach diapedesis-permissive sites [155]. The diapedesis primarily occurs through endothelial junctions (paracellular route) involving endothelial cell junctional molecules including platelet endothelial cell adhesion molecule (PECAM-1), and ICAM-2, CD99 CD99L2 and endothelial cell-selective adhesion molecule (ESAM)-1, JAM family members [155, 156].

The majority of leukocyte TEM is thought to be paracellular, though transcellular migration also occurs, where leukocytes move through the endothelial cell body [157]. Research has shown that prior to endothelial TEM, leukocyte-driven molecular changes induce the clustering of endothelial ICAM-1 and VCAM-1 and the subsequent arrest of leukocytes [158, 159]. The translocation of clustered ICAM-1 to actin- and caveola-rich domains recruits vesiculovascular organelles (VVO) and forms intracellular channels, used for breaching endothelial cells via a transcellular pore [160, 161]. TEM across stimulated endothelial cells of the peripheral circulation occurs largely through the paracellular route (~ 70–90%), whereas brain vascular endothelial cells appear to support a higher rate of transcellular leukocyte TEM [162].

While widely recognised as a critical step in the immune surveillance of tissues, the mechanism through which leukocyte TEM occurs is less well characterised with regards to the role of the ECM [141]. Studies have previously shown that leukocyte-mediated degradation of basement membranes may facilitate their penetration [163]. Yet, given the long half-lives of basement membrane proteins in mature tissues [164], their degradation could lead to long-term alterations in the vascular basement membrane’ [141]. The homeostatic trafficking of T lymphocytes and dendritic cells across the endothelial basement membrane occurs in the absence of irreversible changes to the macromolecular structure of the vascular basement membrane in vivo [165–169]. And, while diapedesis, or the passage of cells through the intact vessel wall, can interfere in the retention properties of the basement membrane, the effects are transient and therefore likely reversible [141]. Nonetheless, the temporal and spatially restricted cleavage of ECM components [170] or their receptors [166] could alter the intermolecular interactions within the basement membrane and facilitate leukocyte extravasation [141].

Leukocyte adhesion to the venular basement membrane may mediate venular wall molecular remodelling, as could occur through interactions of *β* 1 integrin laminin receptors α6*β*1 and α3*β*1 [171, 172]. Accordingly, the transmigration of neutrophils across the venular basement membrane has previously been associated with an increase in the presence of neutrophils carrying venular basement membrane components, including laminin chain isoforms [170, 173, 174]. Through this mechanism, neutrophil transmigration may well give rise to transient alterations to the structure of the venular basement membrane, thereby forming preferential sites for leukocyte extravasation. A further potential mechanism for leukocyte transmigration describes the “thinning” of the venular basement membrane through changes in pericytes and subsequent alterations to tractional force exerted [175, 176]. For example, leukocyte-dependent release of tumour necrosis factor (TNF) may alter vascular permeability through its vasoactive effects, or through signalling to endothelial cells, as may be mediated by ICAM [175, 177]. Finally, the binding of the α4 chain of laminin-411 with type IV collagen may produce a matrix within which the collagen-laminin basement membrane dissociates more readily, potentially mediating leukocyte extravasation [134].

Neutrophils are one of the first responding leukocytes to sites of inflammation, arriving from the blood, transmigrating through the endothelium and the vascular basement membrane in response to infections or injury [178]. The appropriate transmigration of neutrophils is dependent on interactions between cells and the extracellular matrix [179]. Upon recruitment, neutrophils eliminate the encountered threats through the secretion of neutrophil extracellular traps (NETs), which immobilise and kill bacteria [180]. Importantly, the protrusion of NETs from has been observed during their extravasation across the endothelium and vascular basement membrane [181]. Moreover, neutrophils execute their effector functions when immersed within an ECM network [148]. The exposure of cultured neutrophils to laminin 411 and 511 is sufficient to induce NET extrusions in vitro [20, 178] and laminin heterotrimers modulate the chemotactic activity of neutrophils through altering the expression of chemotactic receptors [182–184]. Neutrophil NET expulsion releases neutrophil elastase (NE), cathepsin G and matrix metalloproteinase 9 (MMP9) [185]. The bioactive role of MMP-9 and NE have been implicated in the in vitro remodelling of laminin and aberrant integrin-mediated signalling in cancer cells [185, 186]. Moreover, MMP-9 upregulation may induce BBB breakdown post stroke [187–190]. Intriguingly, treatment of mouse stroke models with a neutralising human IgG monoclonal antibody, L13 (anti-MMP9), significantly reduces brain tissue injury and improves neurological outcomes of mice when administered at the onset of reperfusion [190]. The neuroprotective effects of L13 are directly comparable to the genetic ablation of *Mmp9*, implicating *Mmp9* as a potential target for therapeutic approaches [190]. And, while it remains unclear if MMP-9 specifically cleaves basement membrane laminin during stroke pathogenesis, the involvement of neutrophil-derived proteinases in pathologies affecting the CNS are likely inextricably linked to basement membrane biology.

Investigating the relationships between trafficking immune cells and ECM in vivo provides a clearer understanding of leukocyte extravasation. Post-capillary venules are the site of leukocyte extravasation, and they exhibit ubiquitous laminin 411 and irregular laminin 511 expression, as is seen in the cerebral capillaries [111, 191]. Importantly, impaired recruitment of neutrophils is observed in laminin α4deficient mice [192] whereas T cells [191] and neutrophils [170] extravasate more rapidly at sites of little or no laminin 511 [141] (Fig. 3). Additionally, i*n vitro* analysis of 2D adhesion assays between immune cell populations and laminin 511 revealed inefficient migration capabilities [170]. Furthermore, transgenic mice that ubiquitously express laminin 511 in all endothelial cell basement membranes exhibit a reduction in the infiltration of T cells, macrophages [170] and neutrophils [140] into tissues. Interestingly, comparison of T cell and macrophage infiltration in an EAE model demonstrate that the presence of laminin 511 appears to more potently inhibit the transmigration of T cells, than it does to macrophages [191]. Moreover, in mice with ablated integrin *β1*, *β2*, *β7 *and *α5 *a complete absence of leukocyte extravasation across the endothelial basement membrane of skin postcapillary venules is observed [193]. As such, the ability of immune cells to recognise and traverse preferable sites of extravasation is likely dependent on a variety of factors, either associated with or found within the basement membrane [141].

For example, leukocytes are likely equipped to interact, and signal, with laminin heterotrimers, though laminin-leukocyte signalling cascades are only just beginning to be characterised [194–197]. Binding of T cells to laminins 511 and 411 have been studied using blocking antibodies to integrin-*α*6, -β1 and β3, the laminin-binding integrins expressed on T cells, and arginyl-glycylaspartic acid (RGD) peptides, which block *α*v-integrins [198, 199]. The treatment of T cells with anti-integrin-*α*6 or -β1 reduced their adhesion to laminin 411 by between 50 and 60%, demonstrating a partially redundant role for integrin *α*6β1 in T cell extravasation across the basement membrane. To this end, integrin *α*6β1 and a non-integrin receptor, likely MCAM-1, facilitates T cell-laminin 411 binding, while integrin *α*6β1 and *αv*β1 are required for the adhesion of T cells to laminin 511 [200]. And while encephalitogenic T cells have previously been shown to express integrin *α*vβ3, it is not required for their binding to laminin 511, and is instead required for T cell migration across interstitial matrices, through interactions with vitronectin and fibronectin [201, 202].

The discrete interactions of specific heterotrimers of laminin and T cells is a functionally important step in the pathogenesis of EAE, where encephalitogenic T cells migrate into the brain parenchyma [203, 204]. Indeed, the endothelial basement membrane has been described to act as a checkpoint for entry of pathogenic T during EAE [203]. Mice lacking laminin 411 or 511 show enhanced disease severity due to increased T cell infiltration and altered polarization and pathogenicity of infiltrating T cells [203]. To the best of our knowledge, similar experiments investigating the signalling between other leukocyte subsets and components of the endothelial basement membrane have not been carried out.

Taken together, the mechanisms through which leukocytes penetrate the vascular basement membrane in the CNS remain incompletely defined. However, experimental investigations have revealed that: (A) leukocytes extravasate more readily across the postcapillary venule at sites of low laminin 511 expression; (B) the same type of leukocyte may make use of different mechanisms to penetrate the basement membrane dependent on its composition; (C) the broad proteolysis of basement membrane components is not carried out by transmigrating cells, but selective cleavage of extracellular matrix, or its receptors, may occur; and (D) all transmigrating cell types require integrins or cellular receptors to facilitate the transmigration of the basement membrane [141].

## Basement membrane changes in brain disease

Alterations to the composition of the basement membrane are observed in acute and chronic pathologies of the CNS [205–208]. There is a large body of literature investigating the ECM in response to acute traumatic injury in both brain and spinal cord, particularly in relation to glial scarring and the regulation of neuronal regrowth and is reviewed elsewhere [209, 210]. Here, we use examples of neurological diseases with different aetiologies to highlight how basement membranes are changed and can regulate brain disease.

Globally, stroke is the second leading cause of death and disability and it affects up to one in five people in developed nations and up to almost every other person in low-income countries [211]. Ischaemic stroke is characterised by loss of cerebral blood supply (through clot or haemorrhage) which results in a lesion within the brain parenchyma [212]. Breakdown of the BBB is synonymous with this type of injury [213, 214] and basement membrane degradation has been recorded following ischemia [215, 216]. Basement membrane alterations occur following ischemia as it becomes more diffuse and less electron dense at the ultrastructural levels [217, 218]. And while basement membrane homeostasis exists in a continuum between synthesis and degradation, ischemia appears to enhance matrix metalloproteinase activity [219–224].

Research focusing on the impact of ischemic stroke on basement membrane proteins provides potential mechanisms to understand the cause of basement membrane dissolution. The level of collagen IV decreased in rat brains post stroke across a broad range of ischemic stroke models [225–227]. Experiments investigating the effect of ischemic insult in nonhuman primates demonstrate a significant decrease in the ratio of collagen IV-containing blood vessels in the basal ganglia after ischemia [228]. Whereas, in a murine model of middle cerebral artery occlusion, collagen IV expression remained constant unless an inflammatory challenge, IL-1β, was first used to induce systemic inflammation. Moreover, collagen IV expression has been reported to increase after ischaemia-reperfusion in the spinal cord [229, 230]. Intriguingly, neutrophil infiltration in mice mediate collagen IV loss [172] and collagen IV degradation was shown in a human case study but only in the presence of neutrophil infiltration [231]. Despite this, whether human neutrophils facilitate the degradation of basement membrane collagen-IV post ischemic insult has not been fully characterised. To this end, more research is required to delineate the mechanisms of collagen IV synthesis and degradation in the context of ischemic stroke.

The expression of collagen IV in response to animal models of ischaemia has been reported to increase, decrease or stay stable. These differences may reflect a combination of factors including the use of different species [232], the use of transient or permanent models of occlusion technique to detect collagen IV or brain region assessed. Indeed, antibodies for collagen IV also vary in their immunodetection depending on the method used for sample preparation and antigen retrieval. This technical variation could also explain the observed variability. In addition, collagen IV varies across brain region as vascularisation in the brain parenchyma varies [233] and all these variables must be considered when assessing these data.

Similar findings exist with regards to laminin levels following ischemic stroke. The expression of laminin is significantly downregulated 24 h after reperfusion in mice [234] and ischemia-reperfusion injury in Mongolian gerbils has a similar effect [235]. Investigations involving baboons also demonstrate significant decreases in laminin after transient ischaemia [228]. Interestingly, laminin levels decrease after ischemic stroke in a clinical study involving 50 patients, but its expression appears to revert to baseline by 12 days post stroke [236]. Conversely, laminin remains stable in ischemic brains even with systemic inflammation [229]. Furthermore, significant increases of laminin are found within reactive astrocytes 32 days after ischemia [237]. A transient increase in the expression of laminin is seen in the ischemic brain and in endothelial cells after oxygen-glucose deprivation, mirroring stroke pathology [238]. Of note, many studies have previously employed pan-laminin antibodies to address the role that laminin plays in ischemic stroke pathology, and have not revealed specific roles for specific laminin isoforms [50]. To this end, it is important to investigate specific laminin isoforms in the context of ischemic stroke as changes in their expression patterns are likely intertwined with their biological functions. Indeed, how laminin chain isoform expression patterns change following ischemic injury is fundamental to understand.

The impact of ischemic injury on perlecan and nidogen may also be important for tissue recovery. Perlecan exhibits a 37-61% reduction in expression within two hours following stroke in baboons [205]. Perlecan cleavage is observed in humans and rodent models of ischemic stroke, as is demonstrated through the significant increase in perlecan domain V expression [239, 240]. Perlecan appears to provide a functional benefit in ischemic stroke, as is demonstrated by exacerbations in BBB damage and larger infarct volumes in perlecan-knock out mice [105]. Furthermore, perlecan may promote the recruitment of pericytes through the activity of PDGR*β* and integrin-α5*β*1 [105]. Similarly, the administration of recombinant perlecan domain V appears to reduce infarct volume, attenuate neuronal death, promote angiogenesis, and improve neurological function following ischemic stroke [105, 239, 241, 242]. The expression level of nidogen following ischemic stroke remains poorly investigated. While patients with stroke exhibit increased plasma nidogen levels, a lack of statistical significance has been observed [243]. Intriguingly, exposure of cultured human brain endothelial cells to oxidative stress increases nidogen expression [244]. However, whether the activity of isolated nidogen is comparable to its biological function throughout homeostasis remains unclear.

Alzheimer’s disease (AD) is the most common cause of dementia and occurs because of the atypical accumulation of extracellular amyloid plaques, composed mainly of amyloid beta (Aβ) peptide and intracellular neurofibrillary tangles composed of hyperphosphorylated tau protein [245]. Alterations to the molecular composition of the vascular basement membrane are also implicated in AD pathology [246]. Risk factors for non-familial AD pathogenesis include pathologies affecting the CNS, including ageing, atherosclerosis, stroke, and diabetes [247]. Diabetes, atherosclerosis, and ageing are among pathologies affecting the CNS that have also been associated with changes of the molecular composition of the vascular basement membrane [248–253]. Hence, the exact relationship between alterations to the cerebral vascular basement membrane and AD pathogenesis have been difficult to resolve.

The thickening of the cerebral vascular basement membrane is observed during ageing [246]. However, the capillaries of rodent models of Aβ accumulation and AD patients exhibit exacerbated thickening of the parenchymal basement membrane [207, 208, 254–261]. The affected parenchymal basement membrane shows significant increases in collagen IV content, likely the result of astrocyte or pericyte activity [208, 256, 257, 259]. However, some animal models of AD pathogenesis demonstrate a temporally dependent decline in collagen IV deposition [207, 262, 263]. Whether this discrepancy is the result of varied animal models or continuous, non-uniform changes to the expression levels of collagen IV is not clear. Nevertheless, the thickening of the parenchymal basement membrane is most severe in areas affected by AD neuropathology, implicating a functional consequence [207, 208, 246]. In *vitro* experiments demonstrate that laminins, collagen IV and nidogen disrupt the formation of Aβ -40 and Aβ1−42 fibrils [264, 265]. Conversely, the in vitro interaction of heparan sulfate proteoglycan and α*β *fibrils appears to accelerate and stabilise their formation [266]. Additionally, the significant upregulation of heparan sulfate proteoglycan is observed in the capillaries of AD transgenic mouse models and in postmortem brain samples from AD patients [207, 208, 262]. However, whether basement membrane proteins affect to the stability of Aβfibrils in vivo is not well defined. Whether upregulation of heparan sulfate proteoglycans accelerates amyloid plaque formation remains unclear, but research suggests it is required for beta-amyloid plaque formation [267, 268]. Despite this, collagen IV deposition is upregulated in microvessels of brains from patients with Alzheimer’s disease and hence dysregulation of basement membrane components is implicated in AD pathogenesis [246, 269]. Whether the increased deposition of collagen IV in the vascular basement membrane impedes the clearance of beta-amyloid protein remains unexplored. Increased dysregulation of heparan sulfate proteoglycans and collagen IV clearly influence AD progression, however whether the changes are specific for AD pathogenesis, or an feature of ageing remains undefined [270]. To this end, study of the basement membrane may offer a means to elucidate mechanisms of Alzheimer’s disease progression.

Parkinson’s disease (PD) is another neurodegenerative condition but is characterised by the loss of dopaminergic neurones in the brain with the formation of Lewy bodies, containing aggregated and post-translationally modified *α*-synuclein [271]. Despite the different pathology to AD, PD postmortem CNS tissue also exhibits thickening of the capillary basement membrane [272, 273], and shows string vessels with collapsed basement membranes lacking an endothelium [274]. Collagen IV accumulation in the brain is exhibited by mouse models of PD providing tools to investigate what is seen postmortem in PD patient pathology [272, 273, 275]. Whether thickening of the basement membrane is caused by an increase in the deposition of *α*-synuclein in the vasculature, or whether basement membrane thickening acts in a protective fashion is not known. Given that the alterations to the basement membrane are not seen in all animal models of PD, it remains difficult to assess the functional consequences of the increased collagen IV deposition in human post-mortem tissue. Experiments utilising transgenic mice that overexpress a mutated form of alpha synuclein (A30P) associated with familial PD exhibit upregulated COL4A2 expression [275]. Upregulation of COL4A2 may well interfere with nutrient exchange and thereby potentially contribute to PD pathogenesis, however this remains to be proven. Further investigation into the pathogenesis of PD may yield novel avenues for therapies and interventions.

These examples of brain disease highlight that basement membranes are inextricably linked to damage in the CNS. In a vascular event, basement membranes are affected during ischaemia or haemorrhage (indeed, their dysregulation may have contributed to the ictus), and protection/repair of basement membranes may well be an attractive target for intervention. In neurodegenerative disease, evidence is also mounting that basement membranes are part of pathology, and considering that cerebral small vessel disease [276] and vascular disease contributing to dementia and AD pathology [277] are new frontiers this field, basement membranes importance will grow.

## Summary

The ubiquity and regional specialisation of basements membranes make them fundamental units in tissue homeostasis and therefore a major area of interest for understanding disease. Basement membranes form part of a barrier over which immune cells must cross to during recruitment to injured, infected or diseased tissue. Basement membranes’ major role in leukocyte migration is now appreciated and multiple lines of genetic and experimental evidence show its components are critical for brain health and disruption to them can cause pathogenesis. As such, more fundamental and clinical research into the CNS’s basement membrane will be key in understanding the interaction between the immune and nervous system in health and disease.

## Data Availability

No datasets were generated or analysed during the current study.
